# Dynamic Changes in Oxidative Stress Biomarkers in a Child with Idiopathic Nephrotic Syndrome: A Longitudinal Case Study

**DOI:** 10.3390/ijms27010216

**Published:** 2025-12-24

**Authors:** Joško Osredkar, Matjaž Kopač

**Affiliations:** 1 Institute of Clinical Chemistry and Biochemistry, University Medical Centre Ljubljana, Zaloška Cesta 2, 1525 Ljubljana, Slovenia; josko.osredkar@kclj.si; 2 Faculty of Pharmacy, University of Ljubljana, Aškerčeva 7, 1000 Ljubljana, Slovenia; 3 Department of Nephrology, Division of Paediatrics, University Medical Centre Ljubljana, Bohoričeva 20, 1525 Ljubljana, Slovenia; 4 Faculty of Medicine, University of Ljubljana, Vrazov trg 2, 1000 Ljubljana, Slovenia

**Keywords:** idiopathic nephrotic syndrome, child, oxidative stress, reactive oxygen species, derivatives of reactive oxygen metabolites, plasma antioxidant capacity, oxidative stress index, glucocorticoid therapy, redox biomarkers

## Abstract

Idiopathic nephrotic syndrome (INS) is the most prevalent glomerular illness in children. Even while immunologic processes are well-established, oxidative stress is becoming more widely acknowledged as a significant factor in the etiopathogenesis of illness. Assessing its activity and treatment response may be made easier with the use of trustworthy, non-invasive indicators to track redox balance. We report on the oxidative stress levels of a 10.7-year-old boy with INS with five clinical time points in one year. The FRAS5 analyzer was used to calculate the oxidative stress index (OSI), plasma antioxidant capacity (PAT) and derivatives of reactive oxygen metabolites (d-ROMs) as biomarkers. A 4-tier oxidative state classification scheme based on d-ROM and PAT thresholds was used to interpret the values. The patient had low antioxidant defense, moderate oxidative and increased OSI at relapses, a positive transition to reduced oxidative burden and enhanced defense during remission. The order of events showed a dynamic redox response associated with glucocorticoid (GC) medication and disease activity. The potential value of d-ROM, PAT, and OSI as dynamic biomarkers for tracking disease activity, response to treatment and residual oxidative burden in pediatric INS is supported by this case. To confirm their function in more comprehensive clinical decision-making, more research is required.

## 1. Introduction

The most prevalent glomerular illness in children is idiopathic nephrotic syndrome (INS), which is characterized by widespread edema, hypoalbuminemia, pronounced proteinuria and hyperlipidemia [1]. The disease is chronic and recurrent, and a small percentage of individuals experience glucocorticoid (GC) dependence or resistance, even though the majority of cases respond effectively to GC therapy [2]. Although the exact pathophysiology is yet unknown, evidence points to an immunologically driven breakdown of the glomerular filtration barrier, involving altered charge selectivity of the glomerular basement membrane (GBM) and podocyte damage [3,4].

The most common histological form of INS in children, minimal change disease (MCD), shows effacement of podocyte foot processes on electron microscopy but no discernible structural alterations on light microscopy [5]. Damage to the slit diaphragm and podocyte cytoskeleton increases permeability to macromolecules like albumin [6]. Although glomerular integrity is thought to be disrupted by immunologic triggers, specifically T-cell-derived cytokines, genetic predisposition involving podocyte proteins (e.g., nephrin, podocin) has also been implicated [7].

Oxidative stress has become a crucial cofactor in glomerular damage, surpassing immunological systems [8]. An imbalance between the production of reactive oxygen species (ROS) and the activity of endogenous antioxidant mechanisms leads to oxidative stress. Proteinuria, glomerular injury and active illness have all been linked to elevated lipid peroxidation and impaired antioxidant enzyme activity in INS [9]. Relapses have been linked to biomarkers including malondialdehyde (MDA), advanced oxidation protein products (AOPPs), and decreased glutathione (GSH) and superoxide dismutase (SOD) activity, while remission has been linked to a recovery of antioxidant capacity [10].

While traditional oxidative stress markers provide biochemical insights, newer composite markers offer dynamic, integrative assessments. The circulating hydroperoxides, which are early oxidation products of proteins and lipids, are measured by the derivatives of reactive oxygen metabolites (d-ROM) test and expressed in Carratelli units (U. CARR) [11,12]. Continuous systemic oxidative stress is reflected in elevated d-ROM levels [13]. Simultaneously, the plasma antioxidant test (PAT) measures the blood’s total non-enzymatic antioxidant capacity by reducing ferric ions [14]. A normalized measure of redox balance is the oxidative stress index (OSI), which is computed as the d-ROM/PAT ratio × 100 [15].

These biomarkers have been investigated in metabolic and cardiovascular disorders [15,16,17,18,19], but they are still not very useful in pediatric nephrology, especially when it comes to real-time illness monitoring. Monitoring oxidative stress and recovery in individual patients may help us better understand the dynamics of illnesses like INS and direct treatment.

This longitudinal case report stems from a prospective single-center study including 20 children with idiopathic nephrotic syndrome (INS), in whom oxidative stress biomarkers were measured at predefined disease phases [20]. In that cohort, urinary isoprostanes, serum d-ROMs, and PAT differed significantly across disease stages (*p* = 0.0296, *p* = 0.0458, and *p* = 0.0109, respectively), supporting their relevance as markers of oxidative stress in pediatric INS. Among these children, one boy with a frequently relapsing, steroid-dependent course and complex immunosuppressive management was selected for in-depth longitudinal analysis. He had five well-documented serum sampling timepoints over one year and showed clear temporal coupling between oxidative stress biomarkers and clinical status, including a phase of subclinical relapse. The present report illustrates, through a detailed n-of-1 design, how d-ROMs, PAT, and OSI may dynamically track disease activity and treatment response in a real-world pediatric INS setting.

## 2. Results

### 2.1. Case Presentation

At the study initiation 10.7-years old male patient was admitted to pediatric nephrology department at the age of 9 years due to the first episode of INS with typical clinical presentation consisting of edema, hypoalbuminemia, pronounced proteinuria and hyperlipidemia. He achieved remission after two weeks of GC therapy. Five months later, after discontinuing GC therapy, he experienced his first relapse. He has had a total of ten relapses since then, all of them treated with GC, according to treatment protocols and guidelines. Due to frequent relapses, a kidney biopsy was performed under general anesthesia at the age of 11.8 years. Pathohistological examinations showed minimal glomerular changes. We initiated therapy with mycophenolate-mofetil after kidney biopsy in order to prevent relapses; however, we switched this medication to tacrolimus after six months due to its failure to prevent relapses. Ophtalmological evaluation at the age of 12.2 years revealed decreased vision due to incipient posterior subcapsulary cataract, slightly more prominent in the right eye. He has regular ophtalmological follow-up for this reason.

During the one-year observation period, no major systemic infections (e.g., sepsis, severe pneumonia) or other acute inflammatory illnesses were documented at the times of the five biomarker samplings, reducing the likelihood that the observed redox changes were primarily driven by non-renal intercurrent disease.

The presented patient with INS had five serum samples taken over the course of one year. Clinical milestones, including relapses and remissions were tracked for the patient; however, we failed to catch him in remission post GC treatment, due to frequent relapses.

It is of note that the patient did not receive any drugs at times of relapse, except at relapse confirmation at the start of study period when he received three doses of GC prior to blood sampling, due to technical reasons (start of weekend at examination—1st sample). 3rd sample was taken 20 weeks later and 5th sample 51 weeks after the start of study period. He received only GC treatment at other two blood samplings—at remission (2nd sample—1 week after the start of study period and 4th sample—21 weeks after the start of study period). He began treatment with other immunosuppressive drugs mentioned above (mycophenolate-mofetil and later tacrolimus) several months after the study period and, consequently, after the last blood sampling. These drugs, therefore, could not have influenced the study results.

At long-term follow-up visit, The FRAS5 system was used to measure the blood levels of PAT (plasma antioxidant test) and d-ROM (derivatives of reactive oxygen metabolites) at each time point. The oxidative stress index (OSI) was then computed as (d-ROM/PAT) × 100.

### 2.2. Biomarker Dynamics Across Clinical Timepoints

The oxidative stress profile for each sampling is reported in Table 1. The initial presentation at a relapse revealed a moderately high OSI of 56, decreased antioxidant defense (PAT: 2046 U. CARR) and enhanced oxidative stress (d-ROM: 341 U. CARR). One week later, an OSI of 33 was attained due to a notable decrease in oxidative burden (d-ROM: 235) and a slight improvement in PAT (2271), a change reflected by the patient’s remission achieved by GC treatment. The patient was healthy and without any symptoms or signs of infection or kidney disease at this episode. 20 weeks later relapse was detected, during which the highest OSI value ever recorded (147) was obtained, d-ROM grew significantly to 531 U. CARR, and PAT reached 2437. The patient was placed in oxidative category II according to this pattern, which was consistent with oxidative excess and borderline antioxidant compensation. The patient had only mild, afebrile upper respiratory infection at this time. The blood sample taken one week later showed significant improvement after treatment adjustment (d-ROM: 229; PAT: 2117; OSI: 44). However, the next follow-up 28 weeks later indicated persistent oxidative burden (d-ROM: 422) with a significantly elevated OSI of 92 and a largely unaltered PAT (2081). The patient had only mild diarrhea of one day duration, occurring one day prior to this examination and blood sampling.

**Figure 1:**
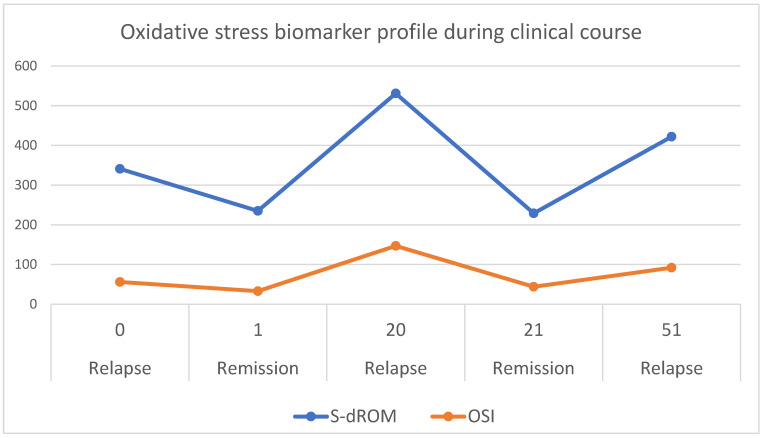
Temporal trends with time points (expressed in weeks of study period) of oxidative stress biomarkers (d-ROM, expressed in U.CARR, and OSI, expressed in relative units) in a child with idiopathic nephrotic syndrome (INS) during study period of one year. Data were collected at five time points corresponding to relapse of a disease (at week 0—start of study period), remission (at week 1), relapse (at week 20), remission (at week 21) and another relapse (at week 51). The d-ROM (blue line) reflects systemic oxidative stress while OSI (orange line) represents the calculated oxidative stress index. Peaks in d-ROM and OSI correspond to disease activity.

Figure 1 illustrates the temporal changes in d-ROM and OSI values across the five clinical stages. The d-ROM and OSI curves show a pronounced peak during relapses, reflecting illness activity: declining during remission and rising during relapse, caused by dynamic balance between oxidative stress and antioxidant defense.

## 3. Discussion

INS development and progression have drawn more attention to oxidative stress, especially in pediatric populations where indirect indicators like serum albumin and proteinuria are frequently used for disease monitoring [1,10,21]. Using d-ROM, PAT, and the derived oxidative stress index (OSI), we longitudinally monitored the oxidative stress dynamics in a child with INS in this case study. We recorded redox changes during five clinical milestones, during relapses and remissions.

In line with an oxidative stress-dominant condition (Situation III), the initial sample at the outset of the disease displayed a reduced antioxidant reserve (PAT: 2046 U. CARR) and moderately enhanced oxidative stress (d-ROM: 341 U. CARR). This is consistent with previous research showing that active nephrotic syndrome is associated with increased levels of protein oxidation and lipid peroxidation [22,23]. The redox sensitivity of these biomarkers was confirmed by the early decrease in d-ROM and the corresponding rise in PAT by the second time point, which indicated a successful early therapeutic response.

Oxidative imbalance significantly increased during relapse, as evidenced by the highest recorded OSI (147) and d-ROM (531 U. CARR). This reversion to Situation II (high oxidative stress, borderline defense) signifies both the reactivation of the disease and the antioxidant system’s inability to make up for it. These results are consistent with research showing increased reactive oxygen species (ROS), decreased glutathione peroxidase (GSH-Px) and enhanced malondialdehyde (MDA) to be associated with relapse episodes [23,24,25].

Both d-ROM and OSI dropped when the patient’s treatment was adjusted and achieved remission, and they returned to Situation IV, indicating a stabilized oxidative balance. At the last long-term follow-up, when mild relapse was detected (clinically silent, however, with nephrotic range proteinuria), d-ROM increased to 422 U. CARR and OSI elevated (92), indicating subclinical oxidative activity despite. Long-term renal failure or increasing podocyte destruction may result from such persistent oxidative pressure; other chronic kidney disorders have also been linked to these phenomena [26,27,28].

Although individual biomarkers, such as MDA or SOD, have been studied in INS before [10,29], using d-ROM, PAT, and OSI together has a number of benefits. First, they can be quickly measured using point-of-care tools like the FRAS5 analyzer and are quantitative and reproducible. Second, the OSI offers a standardized and comprehensive metric that balances antioxidant defense against oxidative burden, a notion that has been validated in the contexts of autoimmune, cardiovascular, and now nephrotic diseases [30,31,32].

This case demonstrates how redox monitoring can supplement traditional clinical evaluations by offering biochemical insights during transitional times (e.g., incomplete remission, steroid weaning). Its potential use in identifying subclinical inflammation or residual disease activity—which may not be visible through standard laboratory testing—is shown by the notable OSI increase during relapse and partial normalization after treatment.

This case does, however, also draw attention to some restrictions. Individual differences in oxidative reactions may restrict generalizability, and the sample size is naturally modest. Furthermore, consistent methodological conditions and reference ranges—which are still lacking in pediatric nephrology—are necessary for the interpretation of OSI.

### 3.1. Limitations and Generalizability

As this is a case report involving a single patient, the generalizability of the findings is inherently limited. While single-case designs provide valuable in-depth insights into individual disease dynamics and serve as important hypothesis-generating studies [33,34], they cannot establish population-level pathological mechanisms or predict treatment responses across diverse patient populations.

This case was selected from our recently published prospective cohort study of 20 children with INS [20], in which we evaluated the same oxidative stress biomarkers across different disease phases. The present patient was chosen for detailed case presentation due to several unique features: (1) exceptionally detailed longitudinal monitoring with five timepoints over one year, (2) frequent relapses (10 episodes total) requiring multiple treatment adjustments, (3) development of steroid-induced complications (posterior subcapsular cataract), and (4) clear temporal correlation between oxidative biomarkers and clinical disease activity.

While our cohort study demonstrated statistically significant changes in d-ROM (*p* = 0.0458), PAT (*p* = 0.0109), and urinary isoprostanes (*p* = 0.0296) across disease phases at the group level, the present case report illustrates how these biomarkers may perform in real-time clinical monitoring of an individual patient. The patterns observed in this case are consistent with our cohort findings, lending biological plausibility to the individual observations.

However, we acknowledge that inter-individual variability in oxidative stress responses, genetic backgrounds, treatment regimens, and disease trajectories limits the direct applicability of these findings to all pediatric INS patients. The observed redox dynamics may be influenced by this patient’s specific clinical characteristics, including steroid dependency and frequent relapsing course.

Future multicenter studies with larger, well-stratified pediatric cohorts are needed in order to (1) establish age-specific reference ranges for d-ROM, PAT, and OSI; (2) validate the predictive accuracy of these biomarkers for relapse prediction; (3) determine optimal monitoring intervals; and (4) assess their utility across different INS phenotypes (steroid-sensitive, steroid-dependent, and steroid-resistant).

### 3.2. Confounding by Treatment Effects and Lack of Controls

A significant limitation of this case report is the absence of control groups, including healthy children and children with other renal diseases. This precludes definitive determination of whether the observed oxidative stress fluctuations are specific to INS pathophysiology or represent effects of pharmacological interventions (glucocorticoids). Several lines of evidence suggest that both disease activity and treatment contribute to the observed redox dynamics:

Evidence for disease-related oxidative stress:

(1) The temporal pattern shows d-ROM peaks during clinically active relapse phases (Week 0: 341 U.CARR; Week 20: 531 U.CARR) with decreases during remission (Week 1: 235 U.CARR; Week 21: 229 U.CARR), suggesting correlation with disease activity independent of treatment timing.
(2) Elevated OSI during subclinical relapse (Week 51: OSI 92, d-ROM 422) occurred before clinician-detected clinical signs, suggesting that oxidative stress reflects underlying disease processes rather than solely treatment effects.
(3) Our published cohort study (n = 20) demonstrated significantly higher d-ROM values at first disease presentation/relapse (before GC initiation in most patients) compared to remission (*p* = 0.0458) [20], supporting disease-driven oxidative burden.

Evidence for treatment-related effects:

(1) PAT values showed patterns suggesting GC-induced modulation of antioxidant defenses. GCs have complex, dose- and duration-dependent effects on oxidative stress: while chronic high-dose exposure can induce oxidative damage through increased mitochondrial ROS production and depletion of antioxidant enzymes [35,36], therapeutic doses may enhance antioxidant gene expression and reduce inflammatory ROS generation [37].

Need for controlled studies:

Distinguishing disease-specific from treatment-induced oxidative changes requires carefully designed studies including healthy age-matched pediatric controls to establish normative ranges; children with other glomerular diseases (e.g., IgA nephropathy, FSGS) to assess INS specificity; comparison of oxidative markers in steroid-naïve vs. steroid-treated patients; longitudinal monitoring during steroid-free remission periods; and correlation with quantitative proteinuria and other disease activity markers independent of treatment.

The lack of untreated controls is an inherent limitation in pediatric nephrotic syndrome research, where withholding effective therapy for observational purposes would be unethical. Future studies should incorporate steroid-free monitoring periods during prolonged remissions to better isolate disease-specific from treatment-related oxidative changes.

### 3.3. Lack of Pediatric Reference Ranges

A significant limitation of this study is the reliance on adult reference values for interpretation of d-ROM and PAT results, as validated pediatric-specific reference ranges are not currently available for these biomarkers. This substantially complicates the interpretation of our findings in a pediatric context and limits clinical applicability.

Current reference values used (adult-derived) are presented in more detail in the Supplementary Materials File S1.

Challenges in pediatric application:

(1) Age-dependent oxidative metabolism: Children exhibit different metabolic rates, growth-related oxidative demands, and antioxidant enzyme maturation compared to adults, potentially affecting baseline d-ROM and PAT values.
(2) Developmental variation: Reference ranges likely vary across pediatric age groups (neonates, infants, children, adolescents), yet age-stratified data are lacking.
(3) Disease-specific considerations: Pediatric INS involves unique pathophysiological features (minimal change disease predominance, higher steroid responsiveness) that may influence oxidative stress profiles differently than adult nephrotic syndrome.
(4) Therapeutic implications: Clinical decision thresholds based on adult values may not accurately identify children at risk for relapse or steroid resistance.

Steps toward pediatric normative data: Our cohort study (n = 20 pediatric INS patients, ages 3–19 years) represents an initial step toward defining pediatric reference ranges [20]. In that study, we observed mean values across different disease phases: d-ROM 240.7–318.7 U.CARR, PAT 2274–2664 µmol/L, and OSI 54.1–71.8. However, these values represent diseased states rather than healthy pediatric controls. Notably, even during remission, our patients’ PAT values (2274 µmol/L) remained below the adult “optimal” threshold (>2800 U.CARR), raising the question of whether this reflects: (a) true antioxidant depletion in pediatric INS, (b) lower physiological baseline in children, or (c) persistent subclinical oxidative stress.

Future directions: Establishment of robust pediatric reference ranges requires large-scale studies in healthy children across age groups; standardization of pre-analytical factors (fasting status, time of day, storage conditions); validation across different analytical platforms and laboratories; and correlation with established pediatric clinical outcomes.

Until such data are available, interpretation of d-ROM, PAT, and OSI in pediatric populations must be performed with caution, focusing on intra-individual trends over time rather than absolute threshold-based clinical decisions. In the present case, we emphasized relative changes across timepoints within the same patient, which minimizes inter-individual variability and provides a more reliable indicator of disease dynamics than comparison to adult normative ranges.

## 4. Materials and Methods

### 4.1. Study Design and Ethical Considerations

This prospective observational case study was conducted at the University Medical Centre Ljubljana. Informed consent was obtained from the patient’s legal guardians, and the study was approved by the National Ethics Committee (Approval No. 0120-501/2022/3, date: 17 January 2023). The study adhered to the ethical principles outlined in the Declaration of Helsinki and relevant local legislation concerning human research.

### 4.2. Clinical Setting and Patient Timeline

The subject was a 10.7-year-old boy with INS, followed over a one-year period through five clinical stages:

1. Initial presentation: mild relapse
2. Remission (early response to therapy)
3. Relapse
4. Remission
5. Relapse

At each stage, clinical evaluation and serum sampling were performed.

### 4.3. Sample Collection

Peripheral blood samples were collected under fasting conditions at each clinical time point. Serum was separated by centrifugation (3000× *g* for 10 min) and immediately processed for oxidative stress analysis using the FRAS5 analytical platform (H&D srl, Parma, Italy). Urine samples were also collected but not used in this specific analysis.

### 4.4. Oxidative Stress Biomarkers

#### 4.4.1. d-ROMs Test (Derivatives of Reactive Oxygen Metabolites)

The d-ROMs test quantifies hydroperoxides—early by-products of oxidative degradation of biomolecules (lipids, amino acids, nucleic acids)—by measuring their ability to oxidize a chromogenic substrate. The results are expressed in Carratelli units (U. CARR), where 1 U. CARR corresponds to 0.08 mg/dL H_2_O_2_ equivalents. Reference values are presented in detail in Supplementary Materials.

#### 4.4.2. PAT (Plasma Antioxidant Test)

The PAT evaluates the non-enzymatic antioxidant capacity of plasma, based on the ability to reduce ferric ions to ferrous form. Results are expressed in U. CARR. Reference ranges are presented in detail in Supplementary Materials.

#### 4.4.3. Oxidative Stress Index (OSI)

OSI was calculated using the formula:

OSI = (d-ROM/PAT) × 100

A higher OSI reflects a disproportionate increase in oxidative stress relative to antioxidant defense, with values over 50 suggesting oxidative imbalance.

### 4.5. Oxidative Status Categorization

Each time point was classified into one of four predefined oxidative status categories based on d-ROM and PAT values, as presented in Supplementary Materials.

## 5. Conclusions

This longitudinal case report suggests that d-ROMs, PAT, and the derived oxidative stress index (OSI) can be feasibly monitored in a child with INS and that, in this patient, their dynamic changes closely paralleled clinical relapses and remissions. The biomarkers demonstrated clear temporal coupling with disease activity, with peaks during relapses and normalization during remissions. Notably, elevated d-ROM and OSI at Week 51 coincided with subclinical relapse (nephrotic-range proteinuria without overt symptoms), suggesting potential utility for early detection of disease reactivation.

While these findings are limited to a single case and require validation in larger, well-stratified pediatric cohorts, they support further evaluation of redox-based markers as adjunctive tools for disease monitoring and treatment tailoring in pediatric nephrotic syndrome. The minimally invasive nature and rapid measurement capability of the FRAS5 system make these biomarkers particularly attractive for longitudinal monitoring in children.

Future multicenter studies are needed to establish age-specific reference ranges; validate predictive accuracy for relapse prediction; determine optimal monitoring intervals; assess utility across different INS phenotypes; and clarify the relative contributions of disease activity versus treatment effects on oxidative stress dynamics.

Despite being restricted to a single case, this study lays the groundwork for further investigations with bigger cohorts to confirm the predictive and surveillance use of redox-based biomarkers in pediatric nephrology. By using these measures, patients at risk of relapse or partial remission could be identified and customized treatment plans could be improved.

## Figures and Tables

**Table 1:** Oxidative stress biomarker profile during clinical progression.

Time Point—Weeks After Start of Study Period (Disease Activity)	d-ROM (U. CARR)	PAT (U. CARR)	OSI	Oxidative Situation	Interpretation
Start of study period (relapse)	341	2046	56	III	Moderate oxidative activity, limited defense
1 (remission)	235	2271	33	IV	Reduced oxidative load, good antioxidant reserve
20 (relapse)	531	2437	147	II	High oxidative stress, borderline defense
21 (remission)	229	2117	44	IV	Low ROS with improved antioxidant status
51 (relapse)	422	2081	92	II–III	Slightly elevated oxidative burden, marginal defense

## Data Availability

The original contributions presented in this study are included in the article/Supplementary Materials. Further inquiries can be directed to the corresponding author.

## Supplementary Materials

The following supporting information can be downloaded at: https://www.mdpi.com/article/10.3390/ijms27010216/s1.

## Abbreviations

The following abbreviations are used in this manuscript:

ROS	reactive oxygen species
MDA	malondialdehyde
SOD	superoxide dismutase
GSH	glutathione
TAC	total antioxidant capacity
TAS	total antioxidant status
GC	glucocorticoid
d-ROMs test	derivatives of Reactive Oxygen Metabolites
PAT	Plasma Antioxidant Test
OSI	Oxidative Stress Index
