# Supplementary material for: Dynamic Changes in Oxidative Stress Biomarkers in a Child with Idiopathic Nephrotic Syndrome: A Longitudinal Case Study
Source: Int J Mol Sci. 2025 Dec 24;27(1):216. doi: 10.3390/ijms27010216 (PMC12785461; doi:10.3390/ijms27010216)
Supplement: Supplementary file 1 [file ijms-27-00216-s001.zip › Supplementary File S1.pdf]

4. Materials and Methods

4.1. Reference Values of Studied Oxidative Stress Biomarkers

4.1.1. d-ROMs Test (Derivatives of Reactive Oxygen Metabolites)

The d-ROMs test quantifies hydroperoxides—early by-products of oxidative degradation of biomolecules (lipids, amino acids, nucleic acids)—by measuring their ability to oxidize a chromogenic substrate. The results are expressed in Carratelli units (U. CARR), where 1 U. CARR corresponds to 0.08 mg/dL H_2O_2 equivalents. Reference values:

- <300 U. CARR: normal oxidative status
- 300–400 U. CARR: borderline oxidative stress
- 400 U. CARR: elevated oxidative stress

4.1.2. PAT Test (Plasma Antioxidant Test)

The PAT test evaluates the non-enzymatic antioxidant capacity of plasma, based on the ability to reduce ferric ions to ferrous form. Results are expressed in U. CARR. Reference ranges:

- 2800 U. CARR: optimal antioxidant capacity
- 2200–2800 U. CARR: moderate antioxidant capacity
- <2200 U. CARR: reduced antioxidant capacity

4.1.3. Oxidative Status Categorization

Each time point was classified into one of four predefined oxidative status categories based on d-ROM and PAT values:

**Situation**	**d-ROM (U. CARR)**	**PAT (U. CARR)**	**Interpretation**
I	275–700	2600–4400	High stress, high defense
II	275–700	1000–2600	High stress, low defense
III	25–275	1000–2600	Low stress, low defense
IV	25–275	2600–4400	Low stress, high defense (ideal redox status)

This classification allowed dynamic assessment of redox status relative to clinical phases (e.g., relapse vs. remission).
